# Drug‐dependent inhibition of nucleotide hydrolysis in the heterodimeric ABC multidrug transporter PatAB from *Streptococcus pneumoniae*

**DOI:** 10.1111/febs.16366

**Published:** 2022-02-11

**Authors:** Charlotte Guffick, Pei‐Yu Hsieh, Anam Ali, Wilma Shi, Julie Howard, Dinesh K. Chinthapalli, Alex C. Kong, Ihsene Salaa, Lucy I. Crouch, Megan R. Ansbro, Shoshanna C. Isaacson, Himansha Singh, Nelson P. Barrera, Asha V. Nair, Carol V. Robinson, Michael J. Deery, Hendrik W. van Veen

**Affiliations:** ^1^ Department of Pharmacology University of Cambridge UK; ^2^ Cambridge Centre for Proteomics Department of Biochemistry University of Cambridge UK; ^3^ Department of Chemistry University of Oxford UK; ^4^ Department of Chemistry University of Cambridge UK; ^5^ Department of Physiology Faculty of Biological Sciences Pontificia Universidad Católica de Chile Santiago Chile; ^6^ Present address: School of Biosciences University of Birmingham UK

**Keywords:** ABC transporter, antibiotic resistance, drug transport, nucleotide hydrolysis, streptococcus

## Abstract

The bacterial heterodimeric ATP‐binding cassette (ABC) multidrug exporter PatAB has a critical role in conferring antibiotic resistance in multidrug‐resistant infections by *Streptococcus pneumoniae*. As with other heterodimeric ABC exporters, PatAB contains two transmembrane domains that form a drug translocation pathway for efflux and two nucleotide‐binding domains that bind ATP, one of which is hydrolysed during transport. The structural and functional elements in heterodimeric ABC multidrug exporters that determine interactions with drugs and couple drug binding to nucleotide hydrolysis are not fully understood. Here, we used mass spectrometry techniques to determine the subunit stoichiometry in PatAB in our lactococcal expression system and investigate locations of drug binding using the fluorescent drug‐mimetic azido‐ethidium. Surprisingly, our analyses of azido‐ethidium‐labelled PatAB peptides point to ethidium binding in the PatA nucleotide‐binding domain, with the azido moiety crosslinked to residue Q521 in the H‐like loop of the degenerate nucleotide‐binding site. Investigation into this compound and residue’s role in nucleotide hydrolysis pointed to a reduction in the activity for a Q521A mutant and ethidium‐dependent inhibition in both mutant and wild type. Most transported drugs did not stimulate or inhibit nucleotide hydrolysis of PatAB in detergent solution or lipidic nanodiscs. However, further examples for ethidium‐like inhibition were found with propidium, novobiocin and coumermycin A1, which all inhibit nucleotide hydrolysis by a non‐competitive mechanism. These data cast light on potential mechanisms by which drugs can regulate nucleotide hydrolysis by PatAB, which might involve a novel drug binding site near the nucleotide‐binding domains.

## Graphical abstract

Heterodimeric ABC multidrug transporters are important contributors to antibiotic resistance in pathogenic bacteria. However, the structural and functional elements determining interactions with drugs and coupling drug binding to nucleotide hydrolysis are not fully understood. Using mass spectrometry and biochemical approaches, we determine the subunit composition of PatAB from *Streptococcus pneumoniae* and probe drug binding locations to explain the inhibition of nucleotide hydrolysis by a subclass of antibiotics and cytotoxic agents.

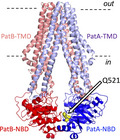

**Abbreviations**
ABC: ATP‐binding cassette
CDL: cardiolipin
DDM: n‐dodecyl‐β‐d‐maltoside
EMA: ethidium‐monoazide
NBD: nucleotide‐binding domain
NBS: nucleotide‐binding site
NTPase: nucleoside‐triphosphatase
TMD: transmembrane domain
TMH: transmembrane helix

## Introduction

ATP‐Binding Cassette (ABC) transporters are ubiquitous membrane proteins responsible for the transport of a variety of substrates, including ions, lipids, peptides, large hydrophobic compounds and a range of pharmaceuticals [1]. Typical bacterial ABC transporters share an overall architecture, particularly at their nucleotide‐binding domains (NBDs) that hold distinct motifs that characterise this protein superfamily [2, 3, 4]. These domains bind and hydrolyse nucleotides and communicate associated conformational changes to transmembrane domains (TMDs) through coupling helices and interconnecting loops. The NBDs contain two composite nucleotide‐binding sites (NBSs) at the dimer interface, which contain conserved sequence elements from both monomers. The Walker A motif is close to the β‐ and γ‐phosphates of bound ATP and contains a conserved lysine residue. The ABC signature sits juxtaposed against the Walker A, sandwiching the ATP molecule in the dimer interface. ATP hydrolysis is thought to be mediated through a catalytic dyad consisting of the Walker B glutamate and the H‐loop histidine [5, 6]. The D‐loop has an important role in NBD dimerisation and allosteric coupling of the NBSs [7, 8]. Further classification splits ABC transporters based on the functionality of the NBSs. In many transporters, both NBSs are active in ATP binding and hydrolysis. However, in the heterodimeric ABC transporters, one NBS contains the classic hallmark sequence motifs responsible for nucleotide binding and catalysis. In contrast, the other NBS possesses a range of mutations in these motifs that allow tight nucleotide binding but disable nucleotide hydrolysis [2, 9, 10].

PatAB is a heterodimeric ABC transporter encoded by two separate genes that are coexpressed in multiple fluoroquinolone‐resistant clinical isolates of Gram‐positive *Streptococcus pneumoniae*, often providing a unique resistance mechanism [11, 12, 13]. When expressed heterologously in *Escherichia coli*, both PatA and PatB monomers are required for the transport of typical substrates of multidrug transporters, such as Hoechst 33342 and ethidium, along with clinically relevant antibiotics [14]. Investigations into the energetics of PatAB have produced interesting discoveries of its preference in nucleotide selection, allowing the hydrolysis of GTP at a faster rate than the classic ATP [15]. Asymmetry at the NBDs leads to NBS1 as a degenerate site containing mutations to the Walker B (D490 rather than consensus E) and H‐loop (Q521 rather than consensus H) of the PatA protein and ABC signature sequence/C‐loop (SAGQ rather than SGGQ) in PatB. The consensus sequences are present for these elements at the canonical NBS2. There is still a lack of understanding of the detailed mechanisms by which the NBSs and transport substrates affect the transport cycle of heterodimeric ABC transporters and why heterodimeric ABC transporters evolved [2, 4, 16]. Using PatAB expressed in Gram‐positive *Lactococcus lactis*, we investigated the interactions of PatAB with a range of antibiotics and cytotoxic agents. The results demonstrate that, unlike homodimeric ABC exporters, nucleotide hydrolysis by PatAB is not stimulated by transported substrates and that nucleotide hydrolysis is non‐competitively inhibited by a subclass of transported substrates. Our analysis of this inhibition introduces a novel model for inhibitory substrate binding at the NBDs of PatAB.

## Results

### PatAB is expressed as a heterodimeric ABC multidrug exporter in *Lactococcus lactis*

**Fig. 1:**
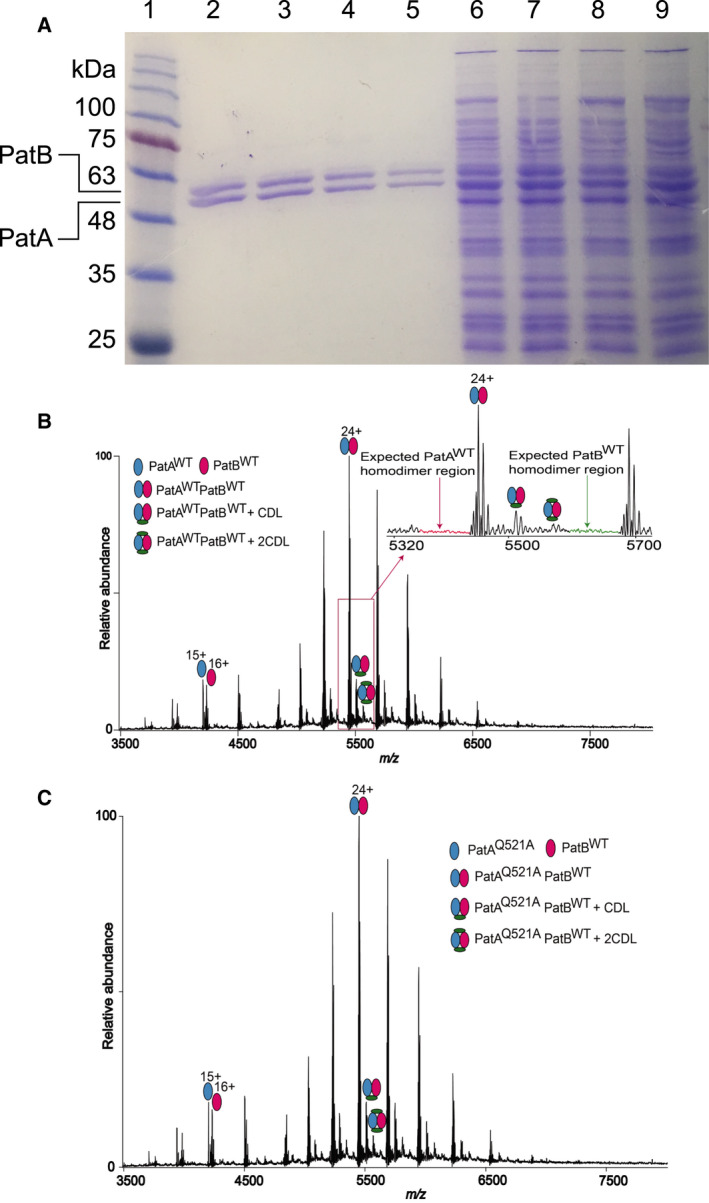
Expression of PatAB proteins in *L. lactis*. (A) Coomassie‐stained 12%‐ SDS/PAGE gel showing affinity‐purified PatA^WT^B^WT^ (*Lane 2*), PatA^Q521A^B^WT^ (*Lane 3*), PatA^WT^B^H543A^ (*Lane 4*) and PatA^Q521A^B^H543A^ (*Lane 5*) and total membrane proteins in lactococcal inside‐out membrane vesicles containing these PatAB proteins (same order in *Lanes 6‐9*). 10 and 15 µg of protein were loaded in *Lanes 2–5* and *Lanes 6–9*, respectively. On the left side of *Lane 1*, the positions of molecular mass markers (kDa) are indicated. (B,C) Native mass spectrum of affinity‐purified PatA^WT^B^WT^ (B) and PatA^Q521A^B^WT^ (C). The analysis shows PatAB heterodimers exclusively without the formation of PatA_2_ and PatB_2_ homodimers. Blue and red ovals refer to PatA proteins and PatB^WT^, respectively. Green ovals indicate lipid binding, consistent with the assignment of one and two cardiolipin (CDL) molecules per complex. Inset in (B) shows the expected regions for PatA_2_ and PatB_2_ homodimer signals, but these signals were not observed.

In this study, N‐terminally His_6_‐tagged wildtype PatA (referred to as PatA^WT^) and C‐terminally His_10_‐tagged wildtype PatB (referred to as PatB^WT^) were coexpressed in Gram‐positive *L. lactis* using the well‐established NICE gene expression system [17]. For this purpose, the *patA* and *patB* genes were PCR amplified from *S. pneumonia*e R6 genomic DNA [18, 19] and cloned into separate pNZ vectors of which one (pNZ8048) contains the standard chloramphenicol resistance marker, and the other (pNZ8048E) has an erythromycin resistance marker [17]. Both plasmids (pNZE‐PatA and pNZ‐PatB) were simultaneously maintained in the lactococcal cells by antibiotic selection. The expression of PatA and PatB in the lactococcal plasma membrane was detected on Coomassie‐stained SDS/PAGE gels as two adjacent protein signals of approx. 60 and 55 kDa (Fig. 1A). When these proteins were excised from the gel, completely digested with chymotrypsin and trypsin, and analysed by liquid chromatography–mass spectrometry (LC–MS/MS), they contained Pat proteins with PatB and PatA sequence coverages of 51% and 38% in the top signal, respectively, versus 40% and 50% in the bottom signal (Data S1). In agreement with the predicted molecular mass for PatB^WT^ (67 kDa) and PatA^WT^ (63 kDa), these results indicate that the top band is PatB^WT^, and the bottom band is PatA^WT^. Densitometric analyses of the PatA^WT^ and PatB^WT^ signals show that these proteins represent approximately 7.2% and 6.7%, respectively, of the total membrane proteins (Fig. 1A).

PatAB complexes were solubilised from the lactococcal plasma membrane with DDM and purified by Ni^2+^–nitrilotriacetic acid (NTA) affinity chromatography according to established protocols [20], and analysed by nano‐electrospray mass spectrometry under native conditions [21, 22, 23] to investigate the subunit stoichiometry, lipid binding and purity of our protein preparations (Fig. 1B,C). For PatA^WT^B^WT^ the mass spectra readily generated abundant charge state series in the mass range of *m/z* 5000–6500 Da centred at 24^+^ charge (Fig. 1B). Deconvolution of this charge state series produced a mass of 130 568 ± 2 Da, which corresponds to the calculated mass of the heterodimer PatA^WT^B^WT^ (130 488 Da). Mass spectra also showed two minor charge state distributions in the mass range *m/z* 3500–5200 Da, centred at 15^+^ and 16^+^ charge states corresponding to monomeric PatA^WT^ and PatB^WT^, respectively, attributed to the presence of monomeric PatA and PatB in solution. Furthermore, the mass spectra showed charge state series with a mass difference of ~ 1420 and ~ 2840 Da from the heterodimer mass. We assign these peaks to the PatA^WT^B^WT^ heterodimer with one or two bound cardiolipin molecules. Charge state distributions corresponding to homodimers of PatA^WT^_2_ and PatB^WT^_2_ were not observed in the mass spectra (Fig. 1B). We conclude that the heterodimer predominates in purified preparations of PatA^WT^B^WT^.

### Ethidium interactions in PatAB

**Fig. 2:**
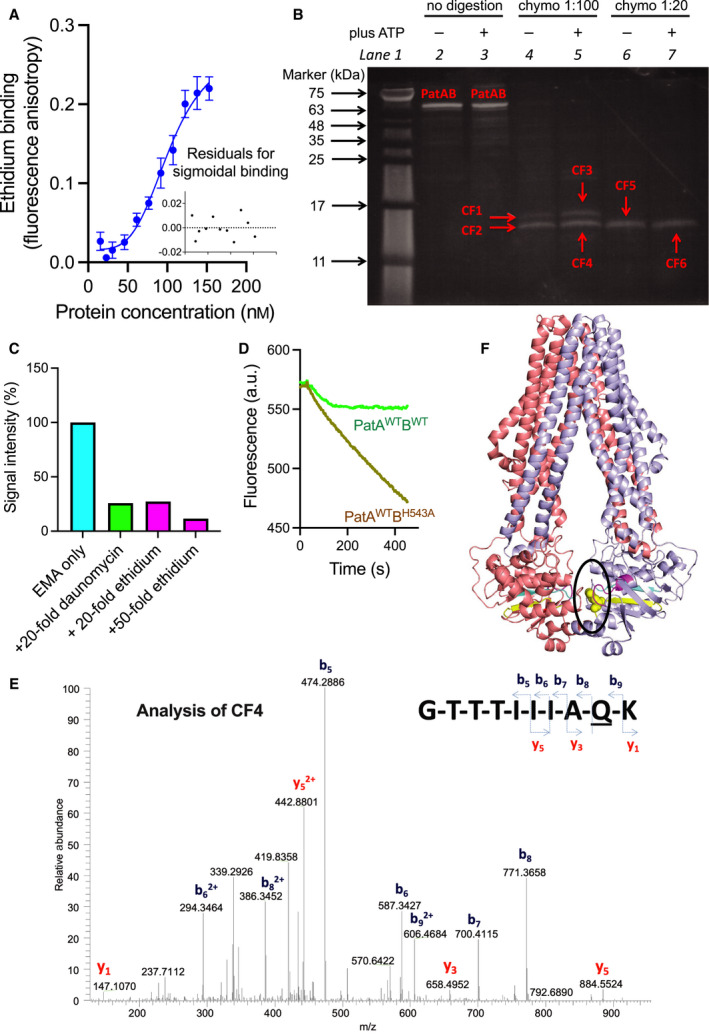
EMA‐labelling of PatAB. (A) Purified PatA^WT^B^WT^ or equivalent volumes of elution buffer were used to titrate 1 μm ethidium, and the fluorescence anisotropy was measured to assess ethidium binding. The changes in anisotropy recorded for the elution buffer were subtracted from those obtained for PatAB protein. Data points represent the mean ± SEM. of three independent experiments (*n* = 3) using different batches of purified proteins. Inset shows the residual variance of the sigmoidal fit. (B) EMA labelling in PatA^WT^B^WT^ and peptides was analysed on a 16% Tricine‐SDS/PAGE gel imaged using in‐gel fluorescence of EMA‐labelled molecules. EMA crosslinking of PatA^WT^B^WT^ in the total membrane protein pool of membrane vesicles in the absence or presence of 0.22 mm ATP was followed by affinity purification (*Lanes 2 and 3*) and partial digestion for 2 h under native conditions by chymotrypsin at chymotrypsin‐to‐protein ratios of 1 : 100 (w/w) (*Lanes 4 and 5*) or 1 : 20 (w/w) (*Lanes 6 and 7*). Separation of the protein digests on the gel showed EMA‐labelled chymotryptic fragments CF1 to CF6 (labelled in red in *Lanes 4–7*). These fragments were completely digested overnight in the presence of trypsin and chymotrypsin at protease‐to‐protein ratios of 1 : 20 and 1 : 10 (w/w) and analysed by LC–MS/MS. On the left side of *Lane 1,* arrows indicate the positions of molecular mass markers (kDa). (C) In‐gel fluorescence of EMA‐labelled PatA^WT^B^WT^ is strongly reduced by incubation with a small 20‐fold excess of daunomycin or ethidium prior to the crosslinking reaction, indicating that EMA binding to PatAB is specific. In‐gel fluorescence was quantified by densitometry using imagej v 1.53j. Shown are representative data from two or more independent experiments with different drug/EMA ratios. (D) Daunomycin is a transported substrate of PatAB. It is effluxed from PatA^WT^B^WT^‐expressing cells in the presence of 20 mm glucose but enters cells expressing the inactive PatA^WT^B^H543A^ mutant, where it intercalates in chromosomal DNA, causing fluorescence quenching. (E) LC–MS/MS spectrum for CF4‐derived peptide identified as 513‐GTTTIIIAQK‐522 from PatA contained the EMA label at the degenerate His‐loop residue Q521. The MS/MS spectrum is generated from the triply charged *m*/*z* 453.24 precursor ion. The representative spectrum is shown from data collected in three independent experiments (*n* = 3). (F) Location of Q521 in the PatA‐NBD in a homology model based on the crystal structure of TM287/288 (PDB‐ID: 4Q4A) and generated in Swiss‐Model. The sequence conservation between PatAB and TM287‐TM288 in the EMBL‐EBI protein alignment tool LALIGN was 39.1% identity (72.2% similar) in a 571‐amino‐acid overlap between PatA and TM287, and 38.1% identity (71.0% similar) in a 583‐amino‐acid overlap between PatB and TM288. The PatA and PatB half‐transporters are shown in light blue and salmon. The Walker A, Walker B and His‐loop sequences in each NBD are shown in magenta, cyan and yellow, respectively. Q521 is presented in yellow spheres within a black circle.

**Fig. 3:**
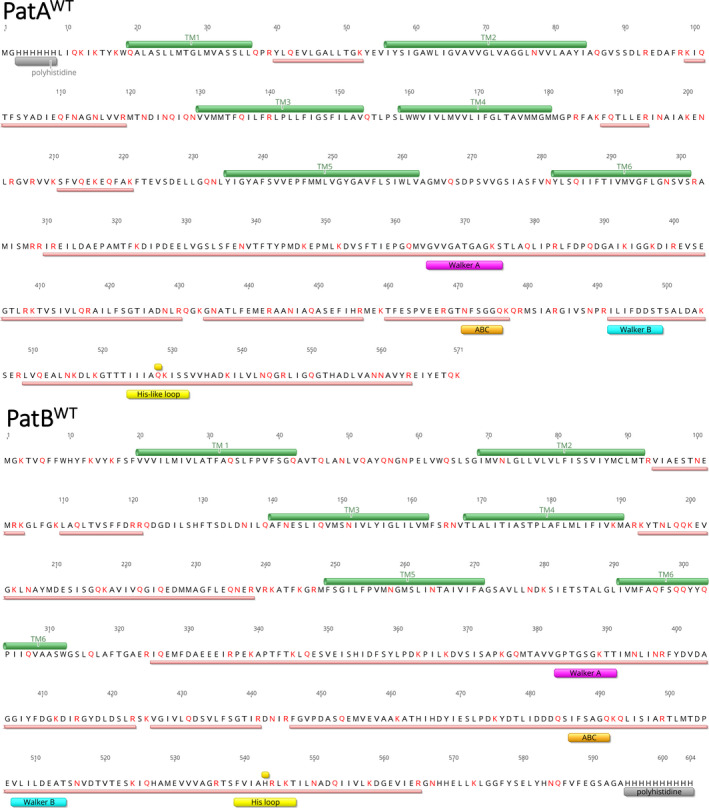
Annotated amino acid sequence of recombinant PatA and PatB. Primary sequences of purified PatA^WT^ and PaB^WT^ recovered from SDS/PAGE in Fig. 1A show predicted membrane‐embedded sections of TMHs (*green cylinders*) and regions recovered in the LC–MS/MS analyses (*salmon rectangles*). K, R, N and Q residues, which are potentially targetted in EMA labelling, are indicated in *red*. These residues are most abundant in the NBDs of PatAB but are also observed in TMH2, TMH3 and TMH6 in PatA and TMH1, TMH3, TMH5 and TMH6 in PatB. Walker A, ABC, Walker B and His‐loop regions are shown in *magenta*, *orange*, *cyan* and *yellow rectangles*, respectively. Figure was generated in Geneious Prime v2022.0.1.

As an established substrate of PatAB [14], the investigation into drug interactions in PatAB focussed on the site(s) of interaction with ethidium (Fig. 2). Equilibrium ethidium binding studies on PatA^WT^B^WT^ based on fluorescence anisotropy measurements revealed sigmoidal binding curves with an average *K*_d_ of 103 nm PatAB and Hill number of 4. These data point to cooperative ethidium binding in PatAB to two or more binding sites (Fig. 2A). These sites were investigated with the photo‐activatable analogue ethidium‐monoazide (EMA) (Fig. 2B–E). Azido‐derivatised substrates have frequently been used in investigations on drug binding in the mammalian ABC multidrug exporters ABCB1, ABCC1 and others [24, 25, 26]. The azido moiety in EMA is highly photosensitive, allowing rapid crosslinking over the course of short bursts of UV light at near‐physiological conditions [24, 27]. In this process, the reactive acryl azide group transforms into a short‐lived nitrene group. The latter can form covalent crosslinks to functional groups containing an electronegative nitrogen atom with a free electron pair [28]. These residues include the side chains of K, R, N and Q, which are most abundant in the NBDs of PatAB but are also observed in the TMDs, in the transmembrane helix (TMH) 2, TMH3 and TMH6 in PatA and TMH1, TMH3, TMH5 and TMH6 in PatB (Fig. 3). The crosslinking of EMA in full‐length PatA^WT^B^WT^ under a 2.5 s UV irradiation burst was performed in lactococcal membrane vesicles in the absence and presence of 0.22 mm Mg‐ATP, and was followed by affinity purification of the transport complex and detection of in‐gel fluorescence on 16% Tricine‐SDS/PAGE (Fig. 2B). Labelling in the presence of increasing concentrations of an alternative substrate resulted in a dose‐dependent fluorescence decrease. The addition of a small 20‐fold excess of ethidium or daunomycin (daunorubicin) over EMA prior to crosslinking was associated with a major 75% reduction in EMA incorporation in PatA^WT^B^WT^ (Fig. 2C). Daunomycin is actively transported by PatA^WT^B^WT^ (Fig. 2D) and other heterodimeric ABC exporters [29]. These results point to the competition between EMA and the PatAB substrates for binding to a defined population of drug binding sites in the transporter [30, 31].

Following crosslinking, PatA^WT^B^WT^ was affinity‐purified and partially digested at chymotrypsin‐to‐protein ratios of 1 : 100 (w/w) and 1 : 20 (w/w) (Lanes 4–7 in Fig. 2B). When run on a 16% Tricine‐SDS/PAGE, the EMA fluorescence was retained in major chymotryptic 15 and 16 kDa signals (labelled CF1 to CF6 in Fig. 2B). As the peptides in these signals were too large for convenient analysis by LC–MS/MS, the chymotryptic signals were excised from the acrylamide gel. The gel fragments were washed and simultaneously exposed to chymotrypsin and trypsin at protease‐to‐protein ratios of 1 : 10 (w/w) and 1 : 20 (w/w), respectively. The small peptides, thus generated in these complete in‐gel digestions, were analysed using LC–MS/MS (Fig. 2E). The PatAB TMDs contain the translocation pathway for drugs across the membrane, and much evidence has been shown for substrate binding in the TMD of other ABC transporters [32, 33, 34]. Therefore, the interaction of EMA with the TMDs was expected. However, no significant EMA‐modified peptides were identified originating from the TMDs, which might relate to the low abundance of such peptides due to the reduced number of K, R, N and Q residues in the TMDs relative to the NBDs, and the incomplete recovery of highly hydrophobic peptides. Despite the low recovery of modified peptides from the TMDs, successful recovery was obtained from in‐gel digestions of chymotryptic fragment CF4. Interestingly, in three independent repeat experiments, the dominant peptide identified, originated from the PatA^WT^‐NBD and matched to region 513‐GTTTIIIAQK‐522 containing an EMA modification of 312 Da on the side chain of Q521 (*n* = 3) (Fig. 2E). Q521 is in the degenerate H‐loop at the equivalent position of H543 in the consensus H‐loop of PatB NBD (Figs. 2F and 3). The reproducibility of the mass spec analysis for CF4 suggests that EMA can bind near the NBDs of PatAB in an orientation at which the azido group becomes covalently attached to the side chain of Q521. Due to the limitations in peptide recovery from the TMDs and the repeatable discovery of the EMA‐crosslinked peptide in CF4, the other chymotryptic CF signals were not subjected to further detailed investigations.

### Ethidium inhibits nucleotide hydrolysis by PatAB proteins

**Fig. 4:**
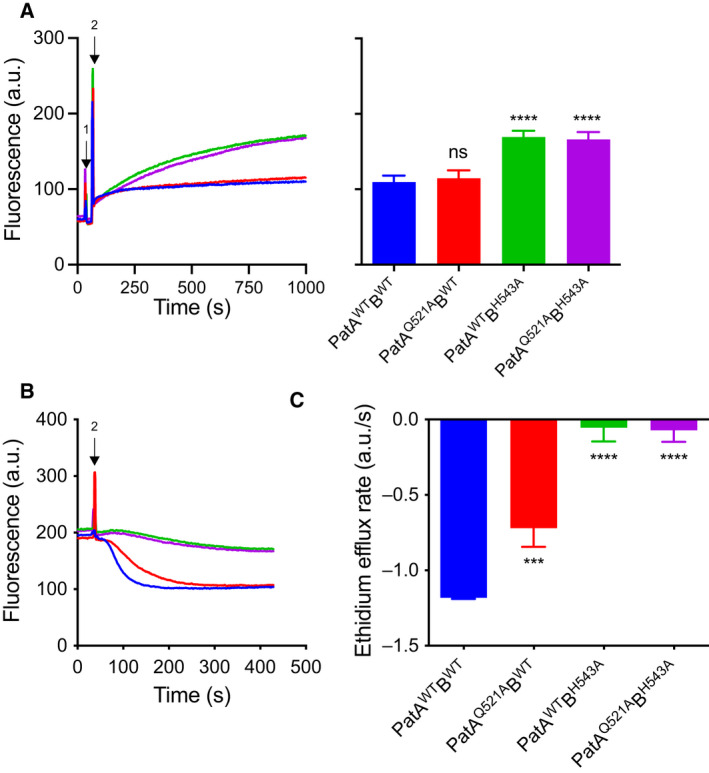
Ethidium transport by PatAB proteins. Ethidium transport was measured in cells expressing PatA^WT^B^WT^ (*blue*), PatA^Q521A^B^WT^ (*red*), PatA^WT^B^H543A^ (*green*) or PatA^Q521A^B^H543A^ (*magenta*). Ethidium intercalation in chromosomal DNA leads to a fluorescence intensity enhancement. (A) For ethidium accumulation, 25 mm glucose was added (*arrow 1*) as a source of metabolic energy, followed by the addition of 2 µm ethidium (*arrow 2*). Histogram represents the mean end‐levels ± SEM. of three independent experiments (*n* = 3). (B) For ethidium efflux, de‐energised cells were exposed to the ethidium and allowed to reach a steady‐state fluorescence at 200 a.u. The cells then received 25 mm glucose (*arrow 2*) to initiate ethidium efflux. (C) The rate of active ethidium efflux after the addition of glucose was calculated from the linear initial slope of fluorescence change in three independent experiments (*n* = 3). Values are expressed as mean ± S.D. (two‐way ANOVA; ***P < 0.001, ****P < 0.0001, ns indicates ‘not significant’ *P* ≥ 0.05). Asterisks refer to comparisons with PatA^WT^B^WT^.

The identification of an EMA binding site in proximity to the degenerate H‐loop region of PatA led to further functional study of the role of degenerate and consensus H‐loop residues in drug transport and ATP hydrolysis. For this purpose, Q521 in PatA and the equivalent H543 in PatB were replaced with alanine and expressed together as PatA^Q521A^PatB^H543A^ or in combination with wildtype half‐transporters, giving PatA^Q521A^B^WT^ and PatA^WT^B^H543A^. These mutants were all equally well expressed in the plasma membrane of the lactococcal cells as PatA^WT^B^WT^ (Fig. 1A). Due to their location at the dimer interface, the dimerisation of the half‐transporters was analysed with native mass spectrometry. Similar to PatA^WT^B^WT^, PatA^Q521^PatB^WT^ showed predominant signals for the heterodimer (Fig. 1B,C). The activity of the mutants was assessed *in vivo* in ethidium transport assays in intact cells. In measurements of ethidium accumulation, the PatA^Q521A^B^WT^ was similarly active as PatA^WT^B^WT^, whereas the mutant PatAB proteins containing the PatB^H543A^ half‐transporter were not transport‐active (Fig. 4A). However, in more sensitive measurements of ethidium efflux, PatA^Q521A^B^WT^ showed a significantly reduced efflux rate compared to PatA^WT^B^WT^ (Fig. 4B,C).

**Fig. 5:**
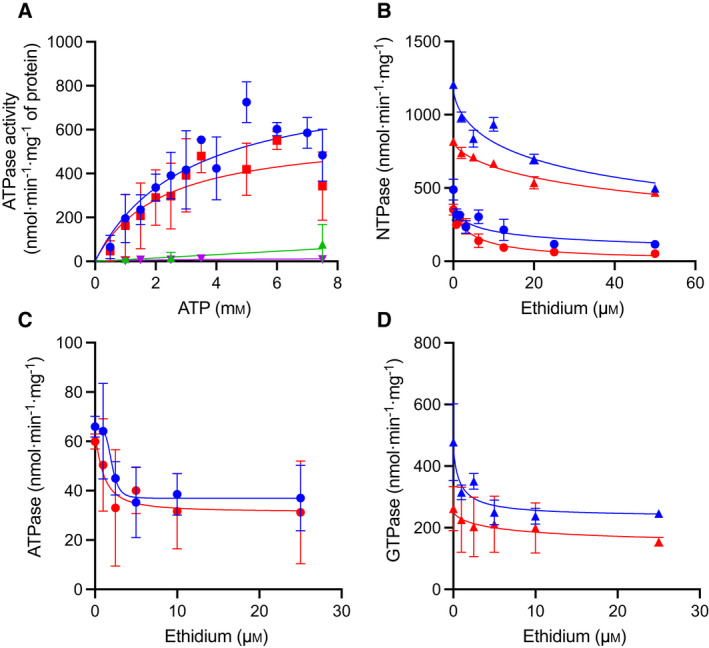
NTPase activity of purified PatAB. (A) Effect of ATP concentration on the rate of ATP hydrolysis by purified PatA^WT^B^WT^ (*blue*), PatA^Q521A^B^WT^ (*red*), PatA^WT^B^H543A^ (*green*) and PatA^Q521A^B^H543A^ (*magenta*) in 0.2% DDM solution (pH 7.0). The solid lines represent the best fit to the Michaelis‐Menten equation. (B) Effect of increasing concentrations of ethidium on the rate of GTP hydrolysis (*triangles*) or ATP hydrolysis (*circles*) shows the inhibition of both PatA^WT^B^WT^ (*blue*) and PatA^Q521A^B^WT^ (*red*) with IC_50_ values as indicated in Table 1. Drugs were premixed with protein before mixing with NTP at a final concentration of 3 mm. (C and D) Ethidium also inhibits ATPase activity (C) and GTPase activity (D) in lipidic nanodiscs. The data points in B–D were fitted to 4‐parameter inhibitor‐response curves and represent the mean ± SEM. (*n* = 5) for detergent and mean ± SEM. (*n* = 3) for lipidic nanodiscs in independent experiments.

PatAB was purified in DDM from the plasma membrane vesicles and used to study the effect of ethidium on PatAB‐mediated nucleotide hydrolysis (Fig. 5). In the absence of drugs, PatA^WT^B^WT^ and PatA^Q521A^B^WT^ exhibited a significant basal ATPase activity, whereas both PatA^WT^B^H543A^ and PatA^Q521A^B^H543A^ were inactive (Fig. 5A). Thus, PatB H543 is critical for nucleotide hydrolysis in PatAB, and, taken with the ethidium transport data (Fig. 4), this hydrolysis is required for efficient transport of the substrate. Conversely, PatA^Q521A^B^WT^ demonstrated a ~ 30% loss in the basal maximum rate (*V*_max_) of ATPase activity relative to PatA^WT^B^WT^ (*V*_max_ = 586 ± 112 nmol Pi min^−1^ mg of protein^−1^ versus 842 ± 111 nmol Pi min^−1^ mg of protein^−1^), whereas the apparent affinity for ATP in the hydrolysis reaction was similar for both (*K*_m_ = 2.2 ± 1.0 versus 3.0 ± 0.9 mm) (Fig. 5A). When taken together, these data demonstrate that the Q521A replacement in the degenerate H‐loop of PatA is associated with a reduction in the rate of the catalytic cycle.

When measured in the presence of ethidium, the *V*_max_ of the ATPase activity was inhibited in a dye concentration‐dependent manner down to approx. 24% of the basal ATPase of the WT protein with a drug concentration giving half‐maximal inhibition (IC_50_) of 7.1 ± 1.8 µm (Fig. 5B and Table 1). Inhibition of the PatA^Q521A^B^WT^ plateaued at approx. 15% of basal ATPase with an IC_50_ of 6.5 ± 1.7 µm. The inhibition of the rate of nucleotide hydrolysis by ethidium was also obtained for GTP, the preferred nucleotide for PatAB under physiological conditions [15] (Fig. 5B), and for purified PatAB in lipidic nanodiscs containing *E. coli* phospholipids and the membrane scaffold protein MSP1D1E3 (Fig. 5C,D). Thus, ethidium inhibition of the rate of nucleotide hydrolysis by PatAB is observed for both ATP and GTP, for protein complexes in detergent solution and when embedded in a phospholipid bilayer.

**Table 1:** IC_50_ values for inhibition by antibiotics of NTPase activity of purified PatAB in detergent solution.

Substrate	IC_50_ (μm)^a^			
	PatA^WT^B^WT^		PatA^Q521A^B^WT^	
	ATP	GTP	ATP	GTP
Ethidium	7.1 ± 1.8	22.7 ± 6.8	6.5 ± 1.7	30.3 ± 9.5
Propidium	0.9 ± 0.6	1.0 ± 0.4	0.9 ± 0.8	2.3 ± 0.6
Novobiocin	4.6 ± 1.7	6.6 ± 1.5	9.7 ± 2.0	6.5 ± 3.3
Coumermycin A1	3.7 ± 0.4	3.1 ± 0.3	3.0 ± 1.0	3.4 ± 0.6

^a^ Data are based on Figs. 5 and 7.

### Inhibition of PatAB NTPase activity by other substrates

**Fig. 6:**
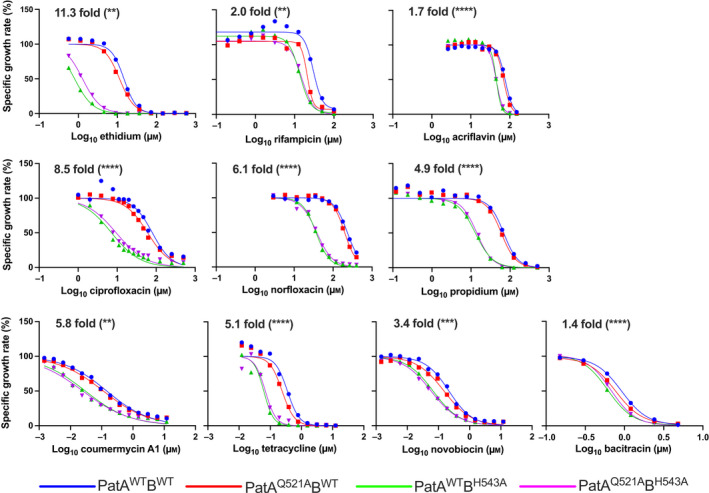
Drug resistance of PatAB‐expressing lactococcal cells. Cell growth was followed at 30 °C by measuring the OD_660_ over time. Impact of increasing concentrations of cytotoxic agents on the specific growth rate of cells expressing PatA^WT^B^WT^ (*blue*), PatA^Q521A^B^WT^ (*red*), PatA^WT^B^H543A^ (*green*) or the catalytically dead mutant PatA^Q521A^B^H543A^ (*magenta*). This rate was calculated by dividing the maximum growth rate in the presence of the drug at the indicated concentrations by the maximum growth rate in the absence of the drug, multiplied by 100%. Data points represent the mean of two to four independent experiments (*n* = 2–4). The IC_50_ for the inhibition of the specific growth rate by drugs was determined by non‐linear fitting to inhibitor‐normalised response curves. Fold difference at the top of the panels refers to the ratio of the IC_50_ values for PatA^WT^B^WT^ and PatA^Q521A^B^H543A^. Asterisks indicate the significance when these IC_50_ values are compared (two‐way ANOVA; **P < 0.01, ***P < 0.001, ****P < 0.0001).

Following the observations of PatAB inhibition by ethidium, the investigations were expanded to a wider range of compounds that are effluxed by PatAB, and hence, are substrates of the transporter. The fluoroquinolones ciprofloxacin and norfloxacin provide good candidates due to the upregulation of PatAB expression in *S. pneumoniae* cells that are exposed to these drugs [11, 13, 18]. Additional antibiotics and fluorescent dyes were also tested but, as direct transport measurements are not possible for most of these molecules, the interaction of these compounds with PatAB was assessed in drug resistance assays in *L*. *lactis* cells overexpressing PatA^WT^B^WT^ or the NBD mutants (Fig. 6). For all tested compounds, specific growth rates showed that PatA^WT^B^WT^‐expressing cells significantly outperformed cells expressing the catalytically dead mutant PatA^Q521A^B^H543A^ as a control (Fig. 6). These drug resistance assays demonstrate that acriflavine, bacitracin, ciprofloxacin, coumermycin A1, norfloxacin, novobiocin, propidium, rifampicin and tetracycline are transported by PatAB. Chloramphenicol and erythromycin were not tested in drug resistance assays due to their use as resistance markers in the lactococcal gene expression vectors but were included in subsequent measurements due to their previously reported transport by PatAB in *S. pneumoniae* [35].

**Fig. 7:**
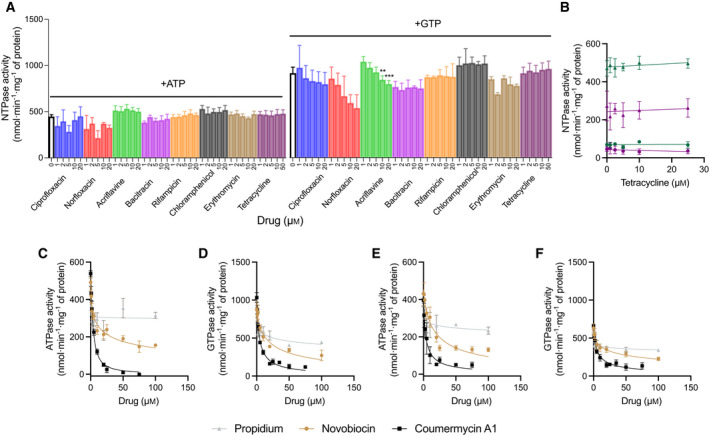
NTPase activity of purified and reconstituted PatAB with transport substrates. (A) Effect of increasing concentrations of drugs (up to 50 µm) on the basal rate of ATP and GTP hydrolysis by PatA^WT^B^WT^ in DDM. Although no effect was seen for most antibiotics, inhibition of the GTPase activity was observed at higher concentrations of acriflavine (Two‐way ANOVA; **P < 0.01, ***P < 0.001, *n* = 3). (B) Lack of inhibition or stimulation of the ATPase activity (*circles*) and GTPase activity (*triangles*) by tetracycline for both PatA^WT^B^WT^ (*green*) and PatA^Q521A^B^WT^ (*magenta*) was also observed in a lipid environment using lipidic nanodiscs. (C–F) Effect of increasing concentrations of drugs on the rate of nucleotide hydrolysis by PatA^WT^B^WT^ (C–D) and PatA^Q521A^B^WT^ (E–F) in detergent solution shows inhibition by propidium (*grey*), novobiocin (*black*) and coumermycin A1 (*gold*). Drugs were premixed with protein before mixing with NTP at a final concentration of 3 mm. The data points in A‐F represent the mean ± SEM. in three independent experiments (*n* = 3). For bacitracin, rifampicin and erythromycin in A, the data are based on two independent experiments.

**Fig. 8:**
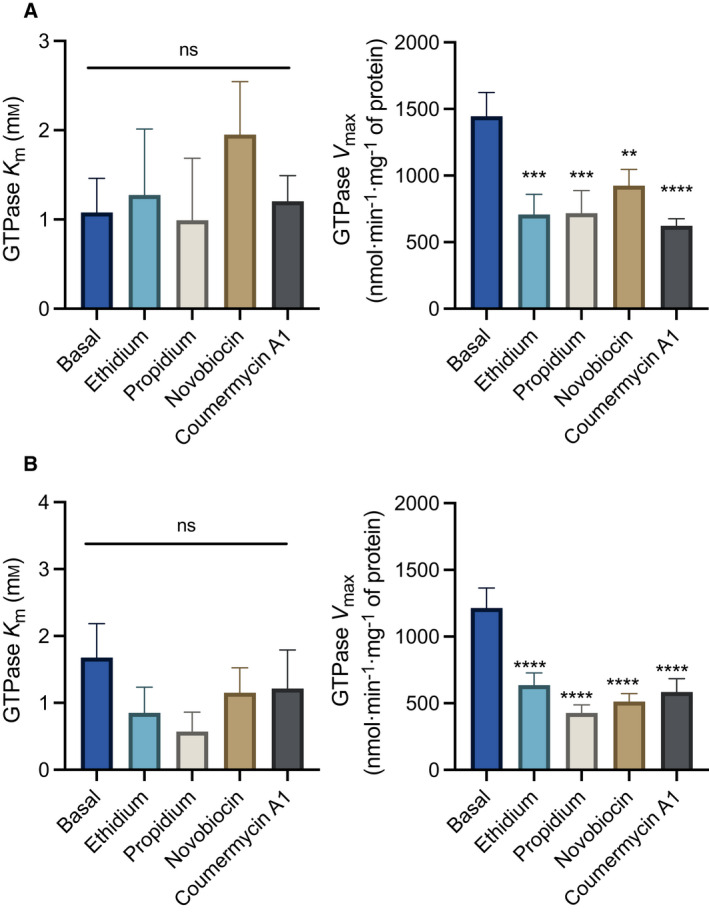
Biochemical mechanism of inhibition of the PatAB GTPase activity by ethidium, propidium, novobiocin and coumermycin A1. Michaelis–Menten parameters for the GTPase reaction by purified PatA^WT^B^WT^ (A) and PatA^Q521A^B^WT^ (B) in DDM in the absence or presence of 5 μm drug. All inhibitory drugs reduced the V_max_ while having no significant effect on *K*_m_, fitting a non‐competitive model for inhibition. Bar heights show the mean ± SEM. in three independent experiments (*n* = 3) (Two‐way ANOVA; ns *P* ≥ 0.05, **P < 0.01, ***P < 0.001, ****P < 0.0001).

The effect of antibiotics and toxic compounds on the NTPase activity was measured for purified PatA^WT^B^WT^ in detergent solution (Fig. 7). Surprisingly, two different types of responses were recorded. First, ciprofloxacin, norfloxacin, bacitracin, rifampicin, chloramphenicol, erythromycin and tetracycline did not cause significant alterations in ATP and GTP hydrolysis by WT protein at drug concentrations of up to 50 µm (Fig. 7A). To confirm that this result was not due to the absence of phospholipids in the protein preparations, the findings were reproduced for tetracycline in lipidic nanodiscs containing PatA^WT^B^WT^ (Fig. 7B). Similar data were also observed in PatA^Q521A^B^WT^‐containing nanodiscs, which shows that the Q521A mutation itself does not alter these drug responses (Fig. 7B). Second, similar to ethidium (Fig. 5), propidium, novobiocin and coumermycin A1 demonstrated significant inhibition of NTPase activity of PatA^WT^B^WT^ and PatA^Q521A^B^WT^ (Fig. 7C–F), with coumermycin A1 being most potent, causing an 85% reduction in the rate of ATP hydrolysis by WT protein with an IC_50_ of 3.7 ± 0.4 µm drug (Fig. 7C and Table 1). Inhibition of GTPase activity gave similar IC_50_ values to the inhibition of ATPase (Fig. 7D,F and Table 1). Even though the fluorescent dye acriflavine had no effect on ATP hydrolysis, it significantly inhibited GTP hydrolysis at the highest concentrations of drug tested (10 and 20 µm) (*P* < 0.01) (Fig. 7A). Thus, mixed first‐and second‐type drug responses were also obtained. To further understand the inhibition of NTPase activity by ethidium, propidium, novobiocin and coumermycin A1, the biochemical mechanisms of inhibition were investigated for the GTPase activity of PatA^WT^B^WT^ and PatA^Q521A^B^WT^ (Fig. 8) which, due to the higher *V*_max_ values, has a better signal‐to‐noise ratio than the ATPase activity in these measurements. The results suggest that these compounds inhibit nucleotide hydrolysis by a non‐competitive mechanism, not significantly altering the *K*_m_ for GTP but significantly reducing the *V*_max_ (Fig. 8).

## Discussion

In this study, we investigated the interactions of the heterodimeric ABC multidrug transporter PatAB with a range of drugs in and purified from lactococcal cells. Native nano‐electrospray mass spectrometry analysis shows that coexpression of PatA^WT^ and PatB^WT^ from two pNZ vectors leads to the formation of a PatA^WT^B^WT^ heterodimer bound to one or two cardiolipin molecules, with no detectable signals for the homodimeric proteins (Fig. 1). With a *K*_m_ of 1.1 ± 0.4 mm and *V*_max_ of 1446 ± 179 nmol Pi min^−1^ mg of protein^−1^ for basal GTP hydrolysis (Fig. 8) and *K*_m_ of 3.0 ± 0.9 mm and *V*_max_ of 842 ± 111 nmol Pi min^−1^ mg of protein^−1^ for basal ATP hydrolysis (Fig. 5), PatA^WT^B^WT^ can hydrolyse these nucleotides with roughly similar apparent affinities but with a faster rate for GTP than ATP. Given the intracellular concentrations of GTP and ATP in the millimolar range in *S. pneumoniae* and the increase in GTP in this bacterium following incubation with PatAB antibiotic substrates [15], these data highlight the physiological preference of PatAB for GTP and ATP as sources of metabolic energy. Interestingly, drugs that can be transported by PatA^WT^B^WT^ had two different effects on the NTPase activity of the purified protein complex. First, for ciprofloxacin, norfloxacin, bacitracin, rifampicin, chloramphenicol, erythromycin and tetracycline, the PatA^WT^B^WT^ complexes in DDM detergent micelles and *E. coli* phospholipid nanodiscs were found to have NTPase activity independent of the drug concentration (Fig. 7A). For these substrates, the NTPase activity of PatAB appears to be always ‘switched on’. Second, increasing concentrations of ethidium, propidium, novobiocin or coumermycin A1 caused progressive non‐competitive inhibition of NTPase activity down to a plateau by reducing the *V*_max_ without affecting the *K*_m_ of the reaction (Fig. 8). As the inhibitor binds to another part of the enzyme than the substrate, non‐competitive inhibition decreases the turnover rate of an enzyme rather than that the reaction is stopped [36].

Our observations on PatAB inhibition raise the question of where drugs bind in this transporter. Without a doubt, the transport of drugs by PatAB will require drug binding to the TMDs, which contain the drug translocation pathways that allow the movement of drugs across the phospholipid bilayer. There is ample evidence for drug binding in the TMDs from functional and structural studies with ABC multidrug transporters [32, 33, 34, 37, 38, 39, 40, 41, 42]. Due to the poor recovery of modified peptides originating from the PatAB‐TMDs, our EMA crosslinking experiments and LC–MS/MS analyses did not resolve ethidium binding in the TMDs. However, our data do suggest further binding of ethidium near the NBDs (Fig. 2), where the azido group of the bound EMA is close enough to the amine moiety of Q521 to crosslink in the degenerate H‐loop of PatA. Drug binding near the NBDs was previously observed in molecular dynamics simulations for the mammalian ABCB1 [43]. Given that novobiocin and coumermycin A1 gave similar inhibitory responses as ethidium in the PatAB NTPase measurements, it is noteworthy that previous studies on the bacteriostatic actions of novobiocin and coumermycin A1 identified the NBDs of DNA gyrase and the related enzyme DNA topoisomerase IV as the target of antibiotic binding [44, 45]. Furthermore, novobiocin has been shown in X‐ray crystallographic studies to bind to the NBD LptB in the *E. coli* lipopolysaccharide ABC exporter LptB2FGC [46, 47]. Substrate‐NBD interactions have also been described for the *E. coli* ABC exporter HlyB with its hemolysin‐A substrate [48]. Finally, the binding of transported substrates near the NBDs is well described for ABC importers. For methionine (MetNI) [49, 50] and molybdate/tungstate (ModBC) [51] transporters, substrate binding at µm concentrations to C‐terminal regulatory domains near the NBDs allosterically inhibits the ATPase activity of the associated NBDs through ‘trans‐inhibition’. In ABC importers, this *trans*‐inhibition mechanism prevents the cytosolic accumulation of transported substrates to unacceptably high levels.

Consistent with non‐competitive inhibition, drug binding near the PatAB NBDs could slow down the nucleotide hydrolysis cycle to a lower level of activity without affecting the nucleotide‐binding affinity. By analogy to the example of novobiocin binding near the NBDs in LptB2FGC, which increases LPS transport through the enhancement of the NBD:TMD interface [46, 47], drug binding near the NBDs of PatAB could increase the NBD–TMD coupling efficiency and reduce the high, potentially partially uncoupled, basal NTPase activity. While H543 in the consensus H‐loop is essential for nucleotide hydrolysis, substitution by A of the equivalent Q521 in the degenerate H‐loop, where we observed EMA labelling, reduces both the NTPase activity and drug efflux rate as detected in drug resistance assays and ethidium transport measurements (Figs. 4, 5, 6, 7, 8) This, along with previously observed higher transport rates for GTP than ATP [15] suggests that NTP hydrolysis and the conformational coupling of this reaction to the TMDs can be rate‐determining steps in the drug transport reaction, giving further support for our suggestion that the basal NTPase activity of PatAB might only be partially uncoupled. Modulation of NBD:TMD coupling has also been suggested for other heterodimeric ABC exporters, where mutations in the degenerate NBS led to the drug‐specific loss or gain in efflux activities [52, 53, 54].

Two other models for drug binding to PatAB should also be considered. First, based on biochemical, biophysical and structural studies, ABC multidrug exporters are known to undergo sequential conformational changes that facilitate drug binding to the TMDs from the cellular interior, followed by drug release from the TMDs towards the external environment. The transport rate is proportional to the fraction of the inward‐facing conformation that has bound the drug and will, therefore, increase with increasing drug concentrations in the cellular interior. However, at the same time, the rate of the reaction will be stimulated by the dissociation of the transported drug from the outward‐facing conformation, which enables the transporter to re‐adopt the inward‐facing state and start a new transport cycle. Consistent with this model, the ATPase activities of homodimeric ABC multidrug exporters, such as Sav1866 and mammalian ABCB1, frequently show bell‐shaped dependencies on the drug concentration, with stimulation of the basal ATPase activity at low drug concentrations but inhibition at high drug concentrations [55, 56]. For PatAB, the basal NTPase activity in detergent solution and lipidic nanodiscs is already maximal and not stimulated by drugs (Figs. 5 and 7). The drugs that show strong inhibition of the NTPase activity (ethidium, propidium, coumermycin A1 and novobiocin) could bind with relatively high affinity to the outward‐facing TMDs compared to drugs that do not inhibit the NTPase activity. This negative feedback of external substrate on the ATPase activity was also suggested for the mammalian antigen ABC transporter TAP1/2 [57] and its bacterial orthologue TmrAB from *Thermus thermophilus* [58]. Second, recent structural data for zosuquidar‐, tariquidar‐ and elacridar‐bound mammalian ABCB1 point to the presence of a voluminous central drug‐binding pocket in which the binding of a single drug at low (nanomolar) concentrations enables its efflux, but the binding of a second drug at higher (micromolar) concentrations can cause an inhibition of conformational changes required for efflux and a reduction in the ATPase activity of the lipid‐reconstituted protein [40, 41]. The sigmoidal ethidium binding to PatAB (Fig. 2A) and NTPase inhibition by ethidium, propidium, coumermycin A1 and novobiocin (Figs. 5 and 7) could also be explained by this ABCB1 model.

As further study into the structures and biochemistry of the ABC family of transporters continues, it is becoming increasingly unlikely that one single model can explain the transport mechanism for all transporters and substrates. Our findings provide evidence for the drug‐dependent inhibition of PatAB NTPase activity in which several mechanisms, including drug binding near the NBDs, can play a role. This latter mechanism (the *cis*‐inhibition of PatAB NBDs) might point to the regulation of PatAB activity by intracellular messenger molecules that can be mimicked by ethidium and other compounds. Further investigations into the locations of drug binding in PatAB will reveal novel aspects of the molecular mechanisms of heterodimeric ABC multidrug exporters and offer new opportunities for the modulation of their drug efflux activities in a clinical setting.

## Materials and methods

### Bacterial strains, cell growth and protein expression

For cloning and site‐directed mutagenesis of the *patA* and *patB* genes, the *E. coli* cloning vector pGEM‐5Zf(+) (Promega, Chilworth, Hampshire, UK) was propagated in *E. coli* XL1‐Blue cells (New England Biolabs, Hitchin, Hertfordshire, UK) grown in Luria‐Bertani Broth medium supplemented with 100 µg·mL^−1^ ampicillin. pGEM‐PatA contained coding regions for an amino‐terminal His_6_‐tag followed by the wildtype *patA* gene (NCBI ID: WP_000908156.1) from *S. pneumonia*e ATCC BAA‐255 / R6 genomic DNA [59]. pGEM‐PatB contained the wildtype *patB* gene (NCBI ID: WP_000859871.1) from this genome with a silenced internal NcoI site (at position 1584) followed by a coding region for a carboxy‐terminal His_10_‐tag. Both gene constructs contained a NcoI site at the 5′ end and an XbaI and SacI site after the stop codon at the 3′ end. Mutant *pat* genes were generated by round‐the‐horn mutagenesis using the following primers: PatA‐Q521A, forward primer 5′‐GCAAAAATTAGCTCGGTTGTCCATGCAGAC‐3′, reverse primer 5′‐AGCAATAATAATGGTTGTCGTCCCCTTC‐3′; PatB‐H543A, forward primer 5′‐GCCCGCTTGAAAACCATTCTCAATGCAGATC‐3′ and reverse primer 5′‐GGCAATGACGAAACTAGTTCTACCTGCTAC‐3′. All genes were sequenced to ensure that only the intended changes had been introduced. The PatAB proteins were expressed in the drug‐hypersensitive *L. lactis* strain NZ9000 Δ*lmrA* Δ*lmrCD* [60] using pNZ vectors and the nisin controlled expression (NICE) system (NIZO food research, The Netherlands) [17]. For this purpose, the wildtype and mutant *patA* genes were subcloned as NcoI/XbaI fragments into linearised pNZ8048E plasmid DNA, containing the erythromycin resistance marker. The *patB* genes were subcloned in a similar fashion in pNZ8048 containing the chloramphenicol resistance marker. The lactococcal cells containing both a pNZ8048 and pNZ8048E‐derived expression vector were grown at 30 °C in M17 medium (Formedium, Hunstanton, Norfolk, UK) supplemented with 25 mm glucose and 3 µg mL^−1^ chloramphenicol and 3 µg mL^−1^ erythromycin. Recombinant expression of wildtype PatA/PatB (PatA^WT^B^WT^), PatA^Q521A^B^WT^, PatA^WT^B^H543A^ and PatA^Q521A^B^H543A^ was induced for 1 h with ~ 10 pg mL^−1^ nisin‐A, through addition of 0.1% (v/v) of the culture supernatant of the nisin producer *L*. *lactis* NZ9700 [17] to cell cultures with an OD_660_ 0.6.

### Preparation of inside‐out membrane vesicles

For the preparation of membrane vesicles, 2 L cultures were harvested at 16 °C by centrifugation (13 000× ***g***, 12 min) after induction of protein expression. Cell pellets were washed once (100 mm K‐HEPES pH 7.0) and resuspended in 40 mL with lysis buffer (100 mm K‐HEPES pH 7.0, 5 mg mL^−1^ chicken egg white lysozyme (Sigma–Aldrich, Merck Life Science UK Ltd., Gillingham, Dorset, UK)) with 1 tablet of Complete‐Protease Inhibitor Cocktail (Sigma–Aldrich). Cells were incubated for 30 min at 30 °C before three passages through a cell disrupter (Basic Z 0.75‐kW Benchtop Cell Disrupter, Constant Systems, Northlands, UK) at 20 000 p.s.i. A further 30 min incubation at 30 °C with 10 µg mL^−1^ DNase and 10 mm MgSO_4_ removed released DNA and RNA. 15 mm K‐EDTA (pH 7.0) was then added, and samples were centrifuged for 40 min (13 000× ***g***, 4 °C) to remove cell debris. Inside‐out membrane vesicles were collected from a final centrifuge (125 000× ***g***, 4 °C) of the resulting supernatant for 1 h. The pellets were resuspended in 50 mm K‐HEPES (pH 7.0) containing 10% (v/v) glycerol, and vesicle suspensions were flash‐frozen and immediately stored in liquid nitrogen.

### Purification of PatAB

Histidine‐tagged PatAB dimers were purified on ice from inside‐out membrane vesicles by Ni^2+^–NTA affinity chromatography. Aliquots of the vesicles were added to solubilisation buffer (50 mm K‐HEPES (pH 8.0), 10% (v/v) glycerol, 0.1 m NaCl and 1% (w/v) n‐dodecyl‐β‐d‐maltoside (DDM)) to a total protein concentration of 5 mg mL^−1^. Solubilisation occurred with gentle mixing at 4 °C for 4–6 h. A 150 µg Ni^2+^‐NTA resin with a binding capacity of up to 50 mg mL^−1^ and bead size between 45 and 165 µm, was pre‐equilibrated by washing 5 times with 5 resin volumes of ultrapure water and twice with 5 resin volumes of wash Buffer A (50 mm K‐HEPES (pH 8.0), 0.1 m NaCl, 10% (v/v) glycerol, 0.2% (w/v) DDM and 20 mm imidazole (pH 8.0)). Solubilised protein was left to bind to washed resin overnight at 4 °C. The resin was collected in 2 mL disposable Biospin chromatography columns (Bio‐Rad, Watford, Hertfordshire, UK) and washed with 5 column volumes of Buffer A followed by 5 column volumes of Buffer B (50 mm K‐HEPES (pH 7.0), 0.1 m NaCl, 10% (v/v) glycerol, 0.2% (w/v) DDM and 20 mm imidazole (pH 8.0)). Bound protein was eluted with 450 µL of Elution Buffer (50 mm K‐HEPES (pH 7.0), 0.1 m NaCl, 10% (v/v) glycerol, 0.2% (w/v) DDM and 150 mm imidazole (pH 8.0)). Protein concentrations were determined with the Pierce Micro BCA protein assay (Thermo Fisher Scientific, Horsham, West Sussex, UK). Purified protein was immediately used for further experiments.

### Native mass spectrometry of purified PatAB transporters

Purified PatAB complexes were analysed by native mass spectrometry following buffer exchange into 200 mm ammonium acetate (pH 8.0) containing 2× the critical micelle concentration of a detergent prepared in‐house (G1) [61] using Biospin columns (Bio‐Rad). 2 µL of ~ 5 µm protein samples were introduced directly into Q‐Exactive UHMR mass spectrometer (Thermo Fisher Scientific) through gold‐coated capillary needles prepared in‐house. The optimised mass spectrometry conditions were as follows: Capillary voltage, 1.2 kV; S‐lens RF, 200%; Trapping gas pressure, 8.0; HCD cell, 150 V; Source trapping, −250V; Resolution, 12 500. Data were analysed using Xcalibur 4.2 (Thermo Fisher Scientific) and unidec [62] software packages.

### Ethidium equilibrium binding to purified PatAB

The fluorescence anisotropy of 1 μm ethidium solution in 2 mL of 50 mm potassium phosphate, pH 7.0, containing 5 mm MgSO_4_ was measured in an LS‐55 fluorescence spectrometer (PerkinElmer, Waltham, MA, USA). Excitation and emission wavelengths were set to 500 and 580 nm, respectively, with slit widths of 10 nm each. Purified protein was diluted to a concentration of 400 μg mL^−1^ and was added to the ethidium solution in a stepwise manner (5 × 2 and 7 × 4 μg). The grating factor was calculated for each addition, and the anisotropy was measured for 1 min with an integration time of 1 s, at 1 min after each addition of protein. Each value is the mean of 6 readings. Elution buffer was used as a negative control. The obtained value for anisotropy was plotted against the concentration of protein and fitted by four‐parameter logistic regression in graphpad Prism v 9.1.2.

### Photoaffinity‐labelling with ethidium monoazide and LC–MS/MS analyses

Inside‐out membrane vesicles containing PatAB were diluted to 5 mg mL^−1^ in photolabelling buffer (100 mm K‐HEPES, pH 7.4, 200 mm KCl and 10 mm MgSO_4_) containing 50 μm ethidium monoazide (EMA) bromide (Invitrogen, Thermo Fisher Scientific). All steps prior to UV exposure were performed in low‐light conditions and black Eppendorf tubes. The reaction mix was incubated for 15 min at 30 °C and then poured onto a plastic petri‐dish (90 mm diameter, height 15 mm). Crosslinking was achieved by irradiation with 150 J of UV (312 nm) at room temperature in a BioLink BLX crosslinker (BDH Laboratory and Scientific Equipment, Merck Life Science UK Ltd.) at 15 cm from five 8‐Watt UV lamps. 1% DDM was then added, and purification was carried out as described in Purification of PatAB.

Purified EMA‐labelled protein was diluted to 0.5 mg mL^−1^ in Elution Buffer. 18 μL of the purified protein was mixed with 2 μL of 0.05 or 0.25 mg mL^−1^ chymotrypsin (Worthington Biochemical Corporation, Lakewood, NJ, USA) on ice to give final chymotrypsin‐to‐protein ratios of 1 : 100 or 1 : 20 (w/w) as required. The mixture was incubated for 2 h at 25 °C. The digest in each reaction mix was terminated by addition of 10 μL of 2× Tricine sample buffer (100 mm Tris–HCl pH 6.8, 8% (w/v) SDS, 24% (v/v) glycerol, 0.02% (w/v) Coomassie Blue G‐250) supplemented with 10 mm DTT and incubated at 37 °C for 15 min. A 15 μL (4.5 μg) of the total mix was then run on a 16% Tricine‐SDS/PAGE gel. After the separation of EMA‐labelled PatAB, bands were visualised under UV light, removed with a scalpel blade and chopped into pieces of approximately 1 × 1 mm. Coomassie Blue stain was removed by washing the gel three times with 100 μL of 50 mm NH_4_HCO_3_/50% HPLC grade acetonitrile each for 10 min at 37 °C. To dehydrate the gel pieces, 50 μL of 50% acetonitrile was added for 10 min at 37 °C and then air‐dried for 20 min. The destaining was followed by a thiol reduction step by incubating the gel pieces with 25 μL of 10 mm DTT/50 mm NH_4_HCO_3_ for 1 h at 37 °C. Protein was incubated with 25 μL of 5 mm iodoacetamide/50 mm NH_4_HCO_3_ in the dark for 45 min at room temperature. Gel pieces were washed twice with 100 μL of 50 mm NH_4_HCO_3_/50% acetonitrile and once with 50 μL of 50% acetonitrile. The gel was then dried for 20 min at room temperature. Finally, the samples were digested overnight with trypsin and chymotrypsin to final protease‐to‐protein ratios of 1 : 20 and 1 : 10 (w/w), respectively, and stored at −20 °C. After digestion, the supernatant was pipetted into a sample vial and loaded onto an autosampler for automated LC–MS/MS analysis.

The sample was analysed by mass spectrometry using a nanoAcquity UPLC (Waters Corp., Milford, MA, USA) system and an LTQ Orbitrap Velos hybrid ion trap mass spectrometer (Thermo Fisher Scientific, Waltham, MA, USA). Separation of peptides was performed by reverse‐phase chromatography using a Waters reverse‐phase nano column (BEH C18, 75 mm × 250 mm, 1.7 mm particle size) at a flow rate of 300 nL·min^−1^. Peptides were initially loaded onto a precolumn (Waters UPLC Trap Symmetry C18, 180 mm × 20mm, 5 mm particle size) from the nanoAcquity sample manager with 0.1% formic acid for 3 min at a flow rate of 10 mL min^−1^. The analytical column was run with Solvent A (water and 0.1% formic acid) and solvent B (acetonitrile and 0.1% formic acid), with a linear gradient of 5% to 35% B for 60 min. The LC eluent was sprayed into the mass spectrometer using a new objective nanospray source. All m/z values of eluting ions were measured in the Orbitrap Velos mass analyser, set at a resolution of 30 000. Ions with charge states of 2+ and above were selected for fragmentation by CID in the linear ion trap. Data were processed using Protein Discoverer v 1.2 (Thermo Fisher Scientific). Briefly, all MS/MS data were converted to mgf files, and these files were then submitted to the Mascot search algorithm (Matrix Science, London, UK) and searched against a custom database, using a fixed modification of carbamidomethyl (C) and variable modifications of oxidation (M), ethidium monoazide and ethidium monoazide + 6 Da (R/N/Q/K/N‐term). Peptides were significant if the reported *P*‐value was <0.05, the precursor ion tolerance was within 100 p.p.m., the fragment ion mass tolerance was within 0.8 Da and the peptide score was 20 or above.

### Drug transport assays

The transport of daunomycin or ethidium was followed in real‐time in *L*. *lactis* cells [63]. 50 mL cultures containing cells with expressed PatAB proteins were harvested by centrifugation at 16 °C (6500× ***g***, 10 min) and washed once with wash buffer (50 mm KPi, 5 mm MgSO_4_, pH 7.0). ATP was depleted in cells by incubation with 0.5 mm 2,4‐dinitrophenol (DNP) at 30 °C for 40 min. Cells were again harvested by centrifugation and washed 3 times with ice‐cold wash buffer to remove any traces of DNP. Cell suspensions were added to 3.5 mL glass cuvettes to an OD_660_ of 0.5 with prewarmed (30 °C) wash buffer to a total volume of 2 mL. Drug (10 µm daunomycin hydrochloride or 2 µm ethidium bromide) was added to the cell suspension, and drug fluorescence levels were monitored in an LS 55B Luminescence Spectrometer (PerkinElmer) at excitation and emission wavelengths of 480 and 590 nm for daunomycin, or 500 and 580 nm for ethidium, each with slit widths of 5 and 10 nm, respectively, to follow drug influx. The addition of 25 mm glucose provided metabolic energy for PatAB‐mediated drug efflux, and fluorescence was monitored for about 10 min. Ethidium transport was also followed in energised cells by the addition of 25 mm glucose to de‐energised cells before the addition of the ethidium.

### Reconstitution of purified PatAB into nanodiscs

Acetone‐ether washed *E. coli* phospholipids and egg‐yolk phosphatidylcholine (Avanti Polar Lipids, Merck Life Science UK Ltd) dissolved in chloroform were mixed in a 3 : 1 ratio (w/w). The solvent was evaporated using N_2_ gas, and the lipid mixture was then rehydrated in Buffer A (50 mm K‐HEPES, 100 mm NaCl, 0.5 mm EDTA (pH 7.5)) to 4 mg mL^−1^ containing 0.19% DDM. Purified PatAB in DDM micelles was mixed with the lipid mixture, MSP1D1E3 (Sigma–Aldrich) and further DDM in the following molar ratios: lipid:MSP1D1E3 (60 : 1), MSP1D1E3 : PatAB (8 : 1) and DDM : lipid (3 : 1) [64]. Mixtures were rocked at 20 °C for 30 min then incubated overnight at 4 °C with rocking. Biobeads SM‐2 (700 mg mL^−1^) (Bio‐Rad) were added to the mixture and incubated for 2 h at 20 °C. Following removal of the Biobeads, the mixture was submitted to affinity chromatography using Ni‐NTA resin prepared with Wash Buffer (50 mm K‐HEPES, 100 mm NaCl, 20 mm imidazole (pH7.5)). Bound protein was eluted with 50 mm K‐HEPES, 100 mm NaCl, 300 mm imidazole (pH 7.0). In‐gel quantification of incorporated PatAB proteins in nanodiscs was performed on 10% Tris‐glycine SDS/PAGE using a range of detergent‐purified proteins of known concentrations as standards. Densitometry of protein bands was performed using li‐cor Image Studio v 5.2.

### NTPase activity of PatAB in detergent solutions and nanodiscs

A colourimetric malachite green assay was used to detect Pi release from ATP or GTP hydrolysis. Malachite green‐ammonium molybdate solution was prepared as previously [60]. Malachite green was filtered and activated with 1 in 100 dilution Triton X‐100 immediately before use. Protein in detergent solution was assayed in a 40 µL reaction mixture containing 50 mm K‐HEPES, 10 mm MgCl_2_, 5% (v/v) glycerol and Na‐ATP or Na‐GTP at pH 7.0 and 30 °C. Reactions were stopped by adding 30 µL of the reaction mixture to 150 µL of Malachite green solution in a 96‐well plate. 40 µL of 34% citric acid was added to stop the reaction after 5 mins of colour development. The OD_600_ was measured 25 min after the addition of the citric acid. For activity assays in the presence of substrate, the substrate was added to the desired final concentrations to reaction buffer before protein and NTP were added. A standard curve was prepared with 30 μL of known concentrations of KPi for each experiment. For protein reconstituted in lipidic nanodiscs, NTPase measurements were carried out under identical conditions in the absence of DDM and glycerol. Curve fitting of NTPase data for determination of kinetic parameters (*K*_m_ and *V*_max_) and IC_50_ values were performed in graphpad Prism v 9.1.2.

### Drug resistance assays

Wildtype or mutant PatAB‐expressing lactococcal cells were grown at 30 °C to an OD_660_ of 0.5–0.6, and then incubated with ~ 10 pg mL^−1^ nisin‐A for 1 h. Subsequently, the cells were diluted to a final OD_660_ of 0.02 in the wells of a 96‐well plate in a prewarmed complete M17 medium containing 10 pg mL^−1^ nisin‐A without or with cytotoxic compounds. Growth was followed for 16 h using a 96‐well Versamax Microplate Reader (Molecular Devices, Wokingham, Berkshire, UK). Growth rates were taken from the exponential phase of cell growth. To calculate IC50 values, the data on specific growth rates versus drug concentrations were fitted to dose–response curves in graphpad Prism v 9.1.2.

### Statistics and reproducibility

The biochemical and biophysical data were obtained using independent batches of cells, lipidic nanodiscs and proteins. The significance of data was tested by two‐way ANOVA. Asterisks directly above bars in the histograms refer to comparisons with control; *P < 0.05; **P < 0.01; ***P < 0.001; ****P < 0.0001; ns, not significant. Specific details are included in the figure legends.

## Conflict of interest

The authors declare that they have no known competing financial or other interests that could have appeared to influence the work reported in this paper.

## Data accessibility

Data that support the findings of this study and DNA and protein sequences under study have been deposited in the University of Cambridge research repository Apollo with DOI link https://doi.org/10.17863/CAM.75508, or are available in the Supplementary Materials of this article or from the corresponding author upon reasonable request. Requests for unique materials should be addressed to the corresponding author. The pNZ plasmids utilise the nisin controlled expression (NICE) system, which was obtained from NIZO Food Research, The Netherlands.

## Author contributions

CG, PYH, AA, WS, ACK, IS, LIC, MRA, HS, AVN and HWVV planned, established methods, expressed mutant proteins, purified and reconstituted PatAB, measured NTPase activities, performed drug resistance, transport and binding assays, and/or generated azido‐ethidium crosslinked proteins and samples for MS. CG generated Figs. 1A and 5. AA, WS, and IS generated Fig. 2A–D. PYH and ACK produced Fig. 4. CG and PYH produced Figs. 6, 7 and 8. JH and MJD planned and performed LC–MS/MS analyses and produced Fig. 2E with protein samples from AA, and generated the Supplementary Materials with samples from MRA. DC, SCI, NPB and CVR planned and performed native MS analyses and produced Fig. 1B,C with protein samples from CG. CG and HWVV wrote the paper with essential input from the other authors in their speciality areas.

## Supporting information


**Data S1**. LC‐MS/MS Mascot search data for top and bottom signal of PatAB on SDS‐PAGE in Fig. 1A.
